# Validity of the EOS-determined pelvic parameters and orientation with pelvic positional variation: a phantom study

**DOI:** 10.1038/s41598-021-89958-y

**Published:** 2021-05-17

**Authors:** Jung-Taek Kim, Dong hoon Lee, Han-Dong Lee, Han-Bit Shin, Bumhee Park, Sunghoon Park, Hyung Keun Song

**Affiliations:** 1 Department of Orthopedic Surgery, Ajou University School of Medicine, Ajou University Medical Center, 164, World cup-ro, Yeongtong-gu, Suwon-si, Gyeonggi-do 16499 Republic of Korea; 2 Donghoon Advanced Lengthening Reconstruction Institute, Seongnam, Republic of Korea; 3 Office of Biostatistics, Medical Research Collaboration Center, Ajou Research Institute for Innovative Medicine, Ajou University Medical Center, Suwon, Republic of Korea; 4 Department of Biomedical Informatics, Ajou University School of Medicine, Suwon, Republic of Korea; 5 Department of Radiology, Ajou University School of Medicine, Ajou University Medical Center, 164, World cup-ro, Yeongtong-gu, Suwon-si, Gyeonggi-do 16499 Republic of Korea

**Keywords:** Experimental models of disease, Preclinical research, Translational research

## Abstract

The EOS is a medical imaging system that incorporates simultaneous orthogonal images, producing three-dimensional (3D) reconstructions of the whole skeletal system in various functional positions. Despite growing interest in the pelvic 3D position, the validity of the EOS has not yet been well studied. We investigated the trueness and precision of EOS imaging for pelvic parameters and orientation and assessed whether the measurement using the EOS was affected by the pelvic orientation itself. The orientation of the anterior pelvic plane and pelvic parameters of a custom-made pelvic phantom were measured by three raters using the EOS, and the measurements obtained were compared with the true values. The standard deviations of the measurement errors were 3.23°, 0.26°, 0.23°, 2.98°, 0.88°, and 3.22° for flexion, obliquity, rotation, pelvic incidence, spinopelvic tilt, and sacral slope, respectively. The root-mean square averages of the standard deviation of each measurement were 4.05°, 0.41°, 0.28°, 4.80°, 0.99°, and 5.13°, respectively. The measurement errors for sacral slope correlated significantly with geometric means of flexion, obliquity, and rotation (r = 0.364, *p* = 2.67 × 10^–11^). The EOS rendered accurate and reliable measurements regarding pelvic 3D position, even with positional variation, but positional variation could affect measurements of sacral slope.

## Introduction

The pelvis plays an important role as a link between the spine and the hips; simultaneously, it serves as a mobile unit both in the spinal column and hip joint. The pelvic incidence, which a morphological parameter of the pelvis, affects positional alignment of the spine^1,2^. To emphasize this concept, Dubousset even referred to the pelvis as the “pelvic vertebra”^3,4^. The acetabular orientation is a determining factor in pathologies of native joints and in complications of replaced joints^5–8^. The acetabulum in the native joint, which is a part of the pelvis, and the acetabular cup in the replaced joint, which is inherently fixed to the pelvis, are influenced by the pelvic orientation^9,10^. With markedly increased interest in the interplay between the spine and the hips, the need for accurate measurements of three-dimensional (3D) pelvic orientation and parameters, in various functional positions of the pelvis, is also growing^11–17^.

Because of its deep location and structural and functional complexity, 3D orientation of the pelvis is difficult to measure. Although conventional radiography and computed tomography have been utilized, both have their limitations. Conventional radiographs, which use a cone-beam X-ray, significantly magnifies the subject to varying degrees^18–20^. The degree of magnification is dependent on the distance of the cassette from the X-ray source, divergence of the X-ray beam, size of the object, and distance from the center of projection field. Moreover, with uniplanar conventional radiography, it is difficult to evaluate the 3D pelvic orientation accurately. Computed tomography (CT) with 3D reconstruction precisely depicts the structure of the pelvis. However, with commonly available CT scanners, the pelvis can be scanned only in the supine position; this prevents evaluation of functional positional parameters. Moreover, a high dose of radiation exposure prevents multiple evaluations in vivo. Although the recently developed upright CT scanners may facilitate evaluation of pelvic orientation and parameters, the current low availability of these upright CT scanners, the large radiation dose, and the inability of these scanners to allow evaluation in functional positions other than in the standing position hamper their use for pelvic evaluation^21^.

The EOS (EOS Imaging, Paris, France) is a biplanar, low-dose X-ray system with two perpendicular fan-shaped X-ray beams and two variable gaseous particle detectors. It can produce full-length, weight-bearing images with 40-fold reduced doses of radiation, i.e., at a fraction of that of plain radiography^22–24^. The SterEOS (EOS Imaging) software is paired with the imaging system, which incorporates simultaneous anteroposterior and lateral images; this makes 3D reconstruction possible at every level of the entire skeletal system^25^. The availability of the EOS has acted as a catalyst for research on 3D pelvic orientation^26–28^.

Several studies have attempted to assess the validity of the EOS previously but most were limited to evaluation of its reliability rather than its accuracy^24,25,29–34^. Studies have evaluated the accuracy of EOS measurements focused on the measurement of the length of the long bones and spinal alignment; however, to the best our knowledge, the accuracy of determining 3D pelvic orientation has been poorly evaluated to date^22,34–36^.

Measurements of pelvic orientation and parameters in simple radiography are known to be affected by the position of the pelvis, as a point source of X-rays is used to make a projection image^37–40^. As measurements on the biplanar stereoradiograph reflect 3D coordinates of the pelvis, the positional effects are expected to be negligible on measurements of pelvic orientation and parameters. Nevertheless, it still uses two projection images for registration of anatomical landmarks, and it remains difficult to control the posture of patients^41^; the validity of measurements of pelvic orientation and parameters could therefore be challenged.

We postulated that the accuracy and reliability of EOS measurements of pelvic orientation and pelvic parameters would be also affected by the orientation of the pelvis itself.

Thus, the purpose of the present study was to determine the (1) trueness and precision of the EOS for pelvic parameters and (2) effect of pelvic orientation itself on the EOS measurement of pelvic orientation and parameters.

## Results

### Trueness (Table 1)

**Table 1:** Trueness of EOS measurements in terms of pelvic parameters and orientation.

	Error margin of positioning device	Rater 1			Rater 2			Rater 3			Total		
		Bias (δ)	MAE	RMEPEM	Bias (δ)	MAE	RMEPEM	Bias (δ)	MAE	RMEPEM	Bias (δ)	MAE	RMEPEM
Flexion	0.14	0.12 ± 1.52	5.11	0.94	− 0.86 ± 5.05	17.26	0.97	− 0.71 ± 1.77	5.00	0.90	− 0.48 ± 3.23	17.26	0.94
Obliquity	0.23	0.19 ± 0.25	0.70	0.50	0.15 ± 0.27	0.70	0.50	0.19 ± 0.26	0.71	0.53	0.18 ± 0.26	0.71	0.51
Rotation	0.19	0.02 ± 0.21	0.50	0.40	− 0.02 ± 0.22	0.59	0.38	− 0.02 ± 0.26	0.63	0.49	− 0.01 ± 0.23	0.63	0.42
Pelvic incidence*		2.44 ± 3.41	9.60		2.44 ± 3.13	9.80		2.27 ± 2.30	7.88		2.38 ± 2.98	9.80	
Spinopelvic tilt	0.14	− 0.09 ± 0.73	3.58	0.75	− 0.48 ± 0.97	3.46	0.88	− 0.57 ± 0.84	2.87	0.90	− 0.38 ± 0.88	3.58	0.84
Sacral slope	0.14	2.53 ± 3.66	10.57	1.00	2.92 ± 3.40	11.22	0.99	2.84 ± 2.51	9.02	0.96	2.76 ± 3.22	11.22	0.98

*RMEPEM* rates of measurement errors out of positioning error margin, *MAE* maximum of absolute error.

*Morphologic parameter.

The bias (δ) values for each measurement are summarized in Table 1. The means of measurement bias for all three gyrations of pelvic orientation and sPT were within the margins of error of the positioning device. The mean of measurement bias for the sacral slope was outside the error margins of the positioning device. The standard deviation of bias for flexion, obliquity, rotation, PI, sPT, and SS was 3.23°, 0.26°, 0.23°, 2.98°, 0.88°, and 3.22°, respectively. Maximal absolute errors of flexion, obliquity, rotation, PI, sPT, and SS were 17.26°, 0.71°, 0.63°, 9.80°, 3.58°, and 11.22°, respectively. All the maximum errors were outside the error margin. The rates of measurement errors outside the positioning error margin (RMEPEM) for flexion, obliquity, and rotation were 0.94, 0.51, and 0.42. The RMEPEM of sPT and SS were 0.84 and 0.98, respectively.

### Precision (Table 2)

**Table 2:** Precision of EOS measurements in terms of pelvic parameters and orientation.

	Rater 1				Rater 2				Rater 3				Overall			
	ICC*	95% CI	RMS_SD_	Global uncertainty	ICC*	95% CI	RMS_SD_	Global uncertainty	ICC*	95% CI	RMS_SD_	Global uncertainty	ICC**	95% CI	RMS_SD_	Global uncertainty
Flexion	0.999	0.998–0.999	1.93	5.37	0.986	0.979–0.990	6.20	17.44	0.998	0.997–0.999	2.60	6.97	0.988	0.983–0.991	4.05	11.33
Obliquity	1.000	0.999–1.000	0.41	1.06	0.999	0.999–1.000	0.42	1.11	1.000	0.999–1.000	0.41	1.07	1.000	1.000–1.000	0.41	1.08
Rotation	1.000	1.000–1.000	0.24	0.68	1.000	1.000–1.000	0.27	0.75	1.000	1.000–1.000	0.34	0.93	1.000	1.000–1.000	0.28	0.79
Pelvic incidence			4.80	13.02			4.20	11.53			3.35	8.99			4.80	12.59
Spinopelvic tilt	1.000	1.000–1.000	0.99	2.72	0.999	0.999–1.000	1.16	3.29	1.000	0.999–1.000	1.11	3.06	0.999	0.999–1.000	0.99	2.87
Sacral slope	0.992	0.989–0.995	5.13	13.91	0.993	0.990–0.995	4.51	12.42	0.996	0.995–0.998	3.56	9.63	0.994	0.992–0.996	5.13	13.47

The ICC of all the parameters revealed excellent reliability (Table 2). The ICC between the measurements of each rater and the reference values showed excellent agreements, with the lower limits of all 95% confidence intervals exceeding 0.95; the ICC among the three raters also had excellent values, approximating to 1.0.

The RMS_SD_ of all the repeated measurements for flexion, obliquity, rotation, PI, sPT, and SS was 4.05°, 0.41°, 0.28°, 4.80°, 0.99°, and 5.13°, respectively. For individual raters, the RMS_SD_ of flexion was the highest among the pelvic orientations, ranging from 1.93° to 6.20°. Among the pelvic parameters, the RMS_SD_ of SS was the highest, ranging from 3.56° to 5.13°.

### Global uncertainty (Table 2)

While flexion was the most uncertain parameter among the pelvic orientations with 11.33° of global uncertainty, SS was the most uncertain parameter among the pelvic parameters with 13.47° of global uncertainty. For individual raters, the global uncertainty of flexion ranged from 5.37° to 17.44° and that of SS ranged from 9.63° to 13.91°.

### Postural influence (Figs. 1 and 2)

**Figure 1:**
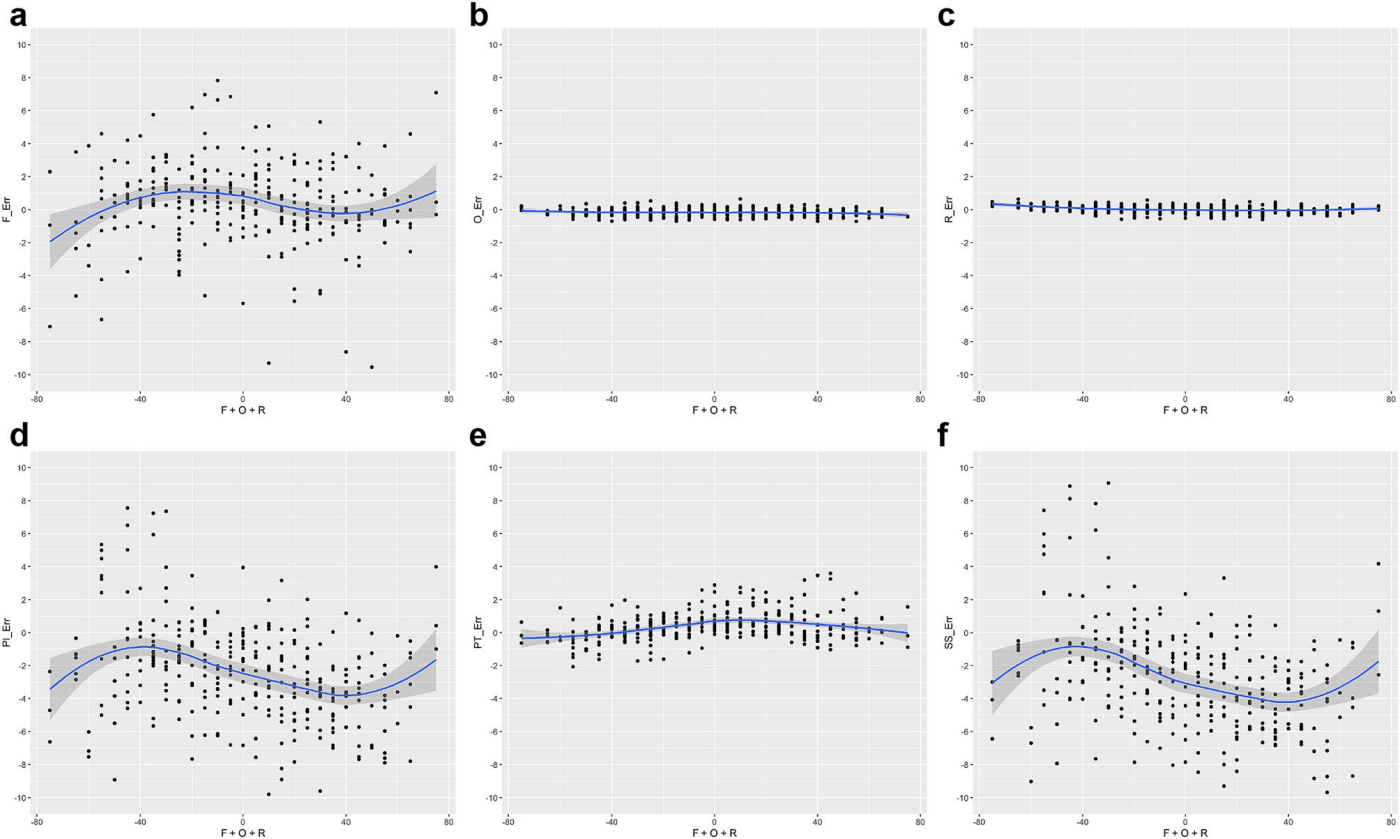
Locally estimated regression graphs for pelvic parameters and orientation. The sum of positional variation values is indicated on the X-axis, and the measurements of each parameter is indicated on the Y-axis. R (version 3.6.1) was used to create the image. (**a**) Flexion, (**b**) Obliquity, (**c**) Rotation, (**d**) Pelvic incidence, (**e**) Spinopelvic tilt, (**f**) Sacral slope.

**Figure 2:**
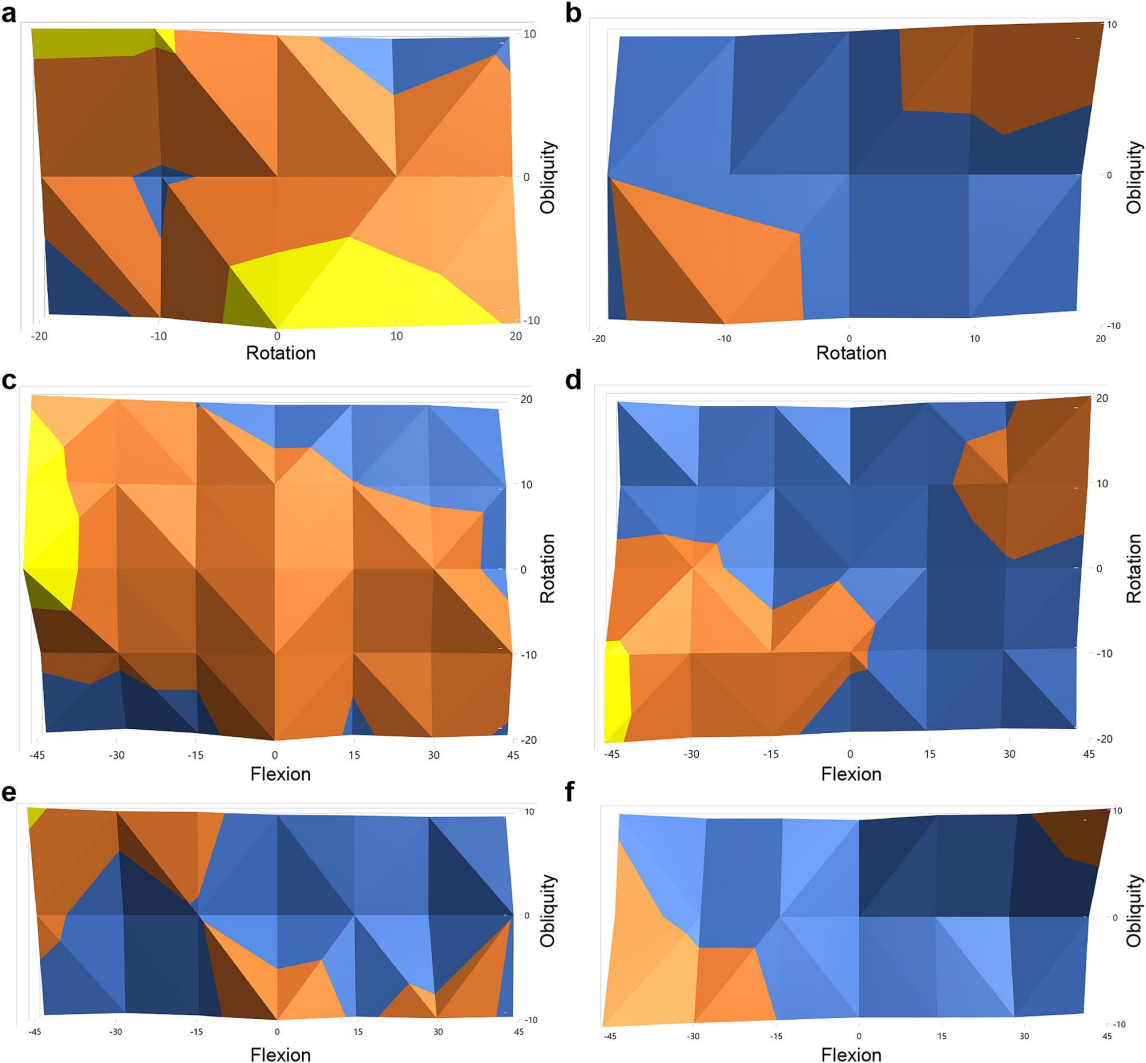
Heat maps for the average measurement errors of the sacral slope were drawn for positional variation in flexion, obliquity, and rotation. Blue indicates the errors in sacral slope ranging between − 7.5° and − 2.5°, scarlet indicates those between − 2.5° and 2.5°, and yellow indicates those between 2.5° and 7.5°. The average measurement errors of sacral slope are shown when the pelvis is positioned as follows: [Microsoft 365 Excel (Microsoft Corporation, Redmond, Washington, USA) was used to create the image. (**a**) In − 45° flexion. (**b**) In + 45° flexion. (**c**) In − 10° obliquity. (**d**) In + 10° obliquity. (**e**) In − 20° rotation. (**f**) In + 20° rotation.

The LOESS plot for measurement errors of each parameters along the sum of pelvic orientation angles depicted that SS and flexion are more error prone than other parameters (Fig. 1). To focus on the measurement errors of these two parameters, we plotted multiple heat maps of measurement errors with positional variance. The heat maps showed that the three positional parameters—flexion, obliquity, and rotation—have a complex impact on measurement errors of SS (Fig. 2).

The heat maps revealed two features of SS measurement errors. First, the sign of SS measurement errors tended to be similar to the sign of the product of flexion, obliquity, and rotation. Second, the absolute values of SS measurement errors correlated with the absolute values of the product of flexion, obliquity, and rotation.

Based on these observations, the correlation between the measurement errors of SS and the geometric means of the positional parameters—flexion, obliquity, and rotation—were tested with Spearman’s correlation analysis, revealing a correlation coefficient of 0.364 (*p* = 2.67 × 10^–11^, 95% CI 0.262–0.463). As PI is the sum of SS and sPT, measurement errors of sPT were relatively smaller than those of SS. Thus, PI was also markedly affected by the geometric means of the positional parameters of flexion, obliquity, and rotation. The coefficient of correlation between PI measurement errors and the geometric means of flexion, obliquity, and rotation was 0.394 (*p* = 3.65 × 10^–13^, 95% CI 0.277–0.489). In contrast to SS, we could not identify any patterns in the measurement errors of flexion.

## Discussion

The present study aimed to measure the accuracy and reliability of the EOS using SterEOS software for measuring pelvic orientation and parameters. The measurements of pelvic orientation and parameters were accurate, with a standard deviation of bias ranging from 0.23° to 3.23°; however, flexion among the pelvic orientations and SS among the pelvic parameters demonstrated the highest measurement errors, with the maximum absolute error reaching 17.26° and 11.22°, respectively. The measurements were reliable, with the average ICC ranging from 0.998 to 1.000. Flexion, among the pelvic orientations, and SS, among pelvic parameters, had the highest RMS_SD_, at 4.05° and 5.13°, respectively. Overall, flexion and SS had the highest global uncertainty, reaching 11.33° and 13.47°, respectively. The geometric mean of flexion, obliquity, and rotation correlated significantly with SS measurement errors (r = 0.364, *p* = 2.67 × 10^–11^).

The limited number of studies available on the validity of the EOS system have focused on the pelvis^22,34,53^. The measurement error of the EOS system for various pelvic orientations was previously assessed by Bittersohl et al^53^. Their analysis was limited in that the positional change did not abide by the standard coordinate system of pelvic orientation^42–44^. Rousseau et al. used a pelvic phantom to assess the effect of axial rotation on the measurement error of the orientation itself. The deviation of axial rotation was − 0.39° ± 0.77°, with a maximal deviation of 1.1°^22^, representing less accuracy and reliability of the EOS system than evaluated in the present study (− 0.01° ± 0.23°, with a maximal deviation of 0.63°). The pelvic phantom used had a unilateral artificial acetabular cup and was not built to make symmetric shape from the designing stage. Without detailed description regarding calibration of rotational axis, a laser indicator to the phantom was attached to the phantom. The inherent asymmetricity of pelvic phantom and dubious spatial calibration may have attenuated the accuracy of reference values in the previous study. Ghostine et al. assessed accuracy only in the neutral position and found that it was less than 1° for pelvic parameters, using synthetic EOS images of a virtual pelvis^34^. All previous studies assessed only the effect of axial rotation rather than 3D positional changes and addressed variation of rotation in the horizontal plane only rather than all components of the 3D positional effect. Studies on clinical images assessed the reliability of pelvic parameters and pelvic orientation measured with the EOS system^24,25,29–34,54^. Studies using clinical images offer greater chances of acquiring study materials during clinical practice and provides valuable information in terms of real-subject variability; however, their scope is limited in that the researcher cannot assess accuracy, as these images lack reference value information.

To overcome the limitations of previous studies, we devised a positioning device to be able to visualize the orientation of the pelvis using the standard coordinate system^42–44^. This allows assessment of measurement errors with complicated pelvis positions, as the pelvis model can be oriented in any direction in 3D space by means of the device (Fig. 6).

The measurement errors obtained with the EOS in the present study were smaller than those reported in studies using radiography^37–40^, indicating that the EOS is one of the most accurate and reliable modalities currently available for the measurement of pelvic orientation and parameters of the pelvis in various functional positions. However, the results of the present study still indicate that correct positioning of patients is required to minimize measurement errors of pelvic parameters and orientation, even with the EOS.

As the EOS imaging system offers comparable accuracy to that of radiostereometric analysis in terms of angular measurements^55^, it may be reasonable to assume that the major source of errors is manual registration of anatomical landmarks^56^.

Even though the EOS uses biplanar stereoradiography to reflect the 3D coordinates of the pelvis, registration of anatomical landmarks is guided on 2D projection images in the SterEOS software^44^.

Not all parameters demonstrated the same level of errors in this study, which agreed with the findings of previous studies^25,29,34,50^. Among pelvic parameters, the measurement of SS was more error prone than that of sPT in the present study. Pelvic flexion was more vulnerable to measurement error than obliquity and rotation of the pelvis. Comparison of the methods of registration used for these measurements may yield insight into the source of the errors.

Measurements of parameters are guided in different ways in SterEOS software^44^. Five anatomical landmarks are involved: (1) the centers of both femoral heads, (2) the center of the upper endplate of S1, (3) the orientation of the upper endplate of S1, (4) the center of both pubic tubercles, and (5) both ASISs. While the measurement of sPT relies on the first two landmarks, measurement of the sacral slope is dependent only on the first and third landmarks^11^. For pelvic orientation, rotation and obliquity only rely on the centers of both femoral heads, while flexion is dependent on the last two landmarks.

Although all anatomical landmarks for registration have round contours, rather than pin-points, the SterEOS uses a point-based registration onto projected 2D images. As the projected 2D images accentuate the tangential surface in the direction of projection, registration onto 2D images can lead to errors^38,57^. As a curve-based method is used for marking the center of the femoral head, the registration was less vulnerable to errors of manual registration^57^. However, registration of the remaining landmarks was guided using a point-based method, which is a heavily error-prone method of registration^57^. Among the remaining measurements, the errors of sPT were smaller than those of SS. To obtain the orientation of the upper endplate of S1, two points located as far as the sacral endplate diameter must be selected, and even small variability in selecting these points may affect the measurements of the S1 endplate orientation^57^. In contrast to the orientation of the upper endplate of S1, the center of the upper endplate of S1 is located far from the midpoint of the centers of both femoral heads. Thus, the measurements of sPT were less affected by variability in selecting the points. Improvement in SterEOS software based on our observations, using 3D reconstructed models for registration and surface-based registration, may further decrease measurement errors.

Our phantom study had several limitations. The present study used a phantom model of a symmetric pelvis. A study of the validity of a specific imaging system requires repetitive acquisition of images; this is not ethically acceptable unless such repeated imaging is clinically required, particularly if the imaging system requires the use of radiation^34^. This issue can be addressed by using a phantom or synthesis of projection images from 3D models^22,34,39^. The pelvis and sacral upper endplate demonstrate large morphometric variations^38,58^. To focus on the effect of position, we used a single symmetric pelvis in the present study. However, similar to the effect of each positional parameter, the shape of an anatomical structure may have complicated effects on the measurements. Moreover, the phantom we constructed lacked soft tissues. In the EOS images acquired in clinical practice, complex contours or markings of soft tissue can have confounding effects on measurements, causing additional measurement errors. Constructing a phantom with a soft tissue is technically demanding, and it is rarely reported in the literature^39,40^. However, constructing a phantom with a soft tissue mounted on the positional device implemented in the present study may overcome this limitation.

## Conclusion

The EOS imaging and measuring system rendered accurate and reliable information regarding pelvic orientation and pelvic parameters, irrespective of positional variation. However, positional variation can differently affect the measurements of pelvic flexion and the sacral slope.

## Methods

This was an experimental study, utilizing a custom-made pelvic phantom. A phantom is a surrogate object that simulates body parts of patients for medical research or calibration of medical devices; the phantom was mounted on a positioning device. The study was approved by the Ajou University Institutional Review Board of our hospital (AJIRB-MED-DEV-19-471). In the present experiment, no human participant other than CT scan images were involved. The requirement for informed consent was waived by the Institutional Review Board of our hospital as performing CT was part of patient’s healthcare and the use of these data posed minimal risk to the patient. All methods were performed in accordance with the relevant guidelines and regulations.

### Pelvic orientation

A globographic coordinate system was used to describe the 3D orientation of the pelvis^42–44^.

**Figure 3:**
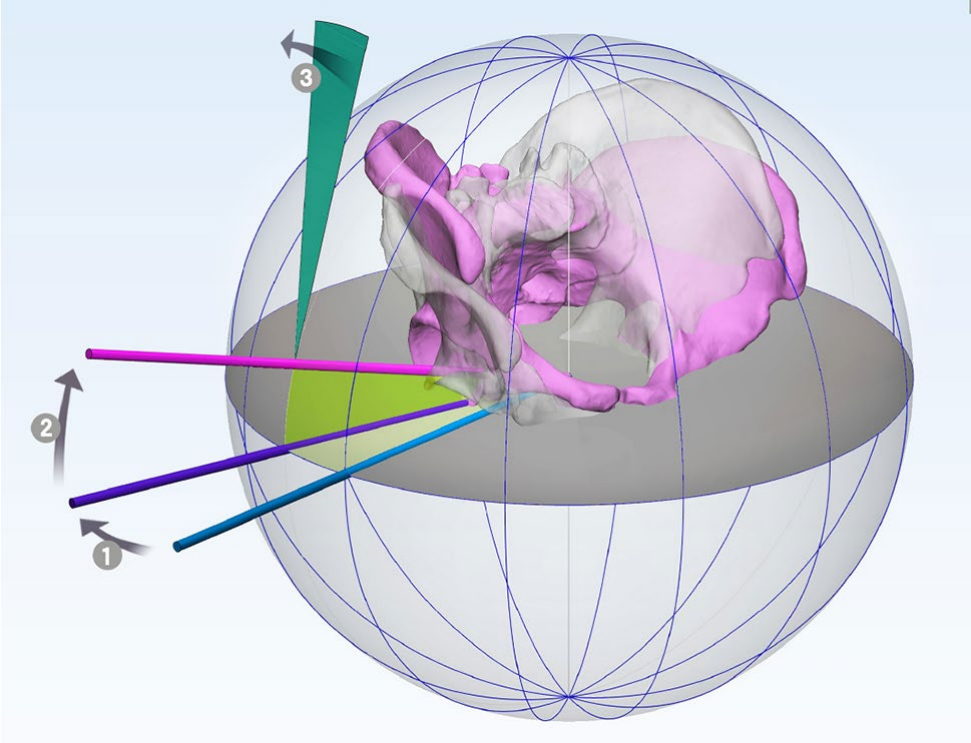
Pelvic orientation described according to the globographic coordinate system. The sequence of rotation (1), obliquity (2), and flexion (3) agrees with conventional clinical understanding^42,43^. The transparent and grey pelvis indicate the neutral position, while the opaque and pink pelvis indicate the position with − 15° rotation, + 15° obliquity, − 15° flexion from neutral position (the direction of +/− is described in the text). MIMICS 20.0 (Materialise, Leuven, Belgium) and 3-matic 12 (Materialise) were used to create the image.

**Figure 4:**
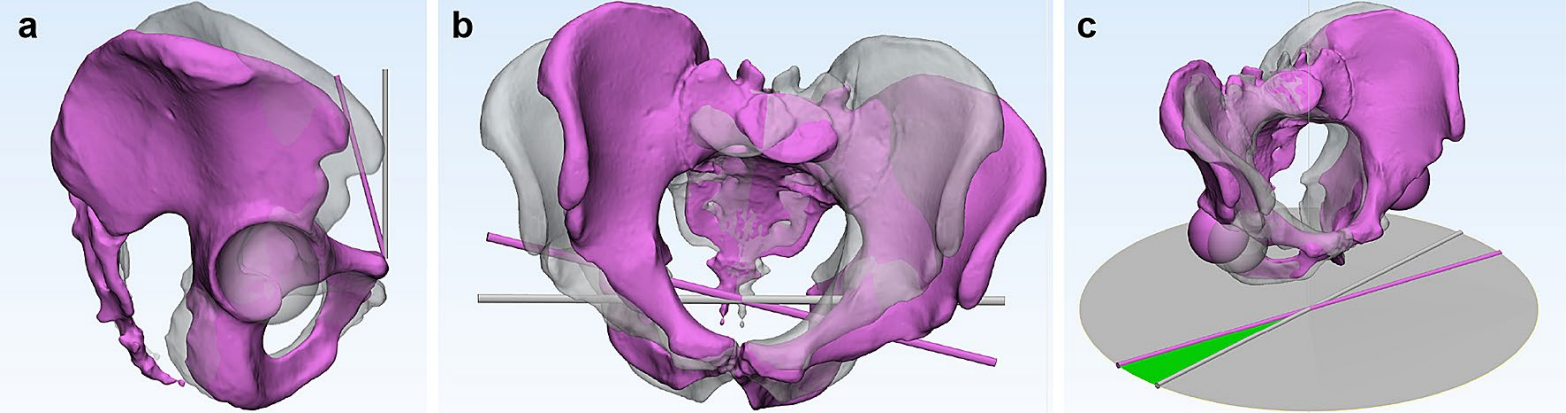
Representation of 3D pelvic orientation. The transparent grey pelvis indicates a neutral position, while the opaque pink pelvis indicates the position with each gyration. MIMICS 20.0 (Materialise, Leuven, Belgium) and 3-matic 12 (Materialise) were used to create the image. (**a**) Flexion: an example of − 15° flexion, (**b**) Obliquity: an example of + 15° obliquity, (**c**) Rotation: an example of − 15° rotation.

In brief, the pelvic rotation angle was defined as the angle between the frontal radiographic plane and the projection of the bicoxofemoral axis to the horizontal plane. Positive rotation corresponded to displacement of the symphysis in the left acetabular direction (clockwise rotation from the distal view). The pelvic obliquity angle was defined in the pelvic frontal plane as the angle between the bicoxofemoral axis and the horizontal plane. A positive value indicated that rotation had occurred in the clockwise direction when observed from the front. The pelvic flexion angle was defined in the pelvic sagittal plane as the angle between the pelvic frontal plane and the anterior pelvic plane, which is a plane formed by both anterior superior iliac spines and the center of the pubic tubercles. Positive flexion corresponded to displacement of the symphysis in the caudal direction (Figs. 3 and 4). The angles of these three gyrations defined the 3D orientation of pelvis.

### Pelvic parameters

**Figure 5:**
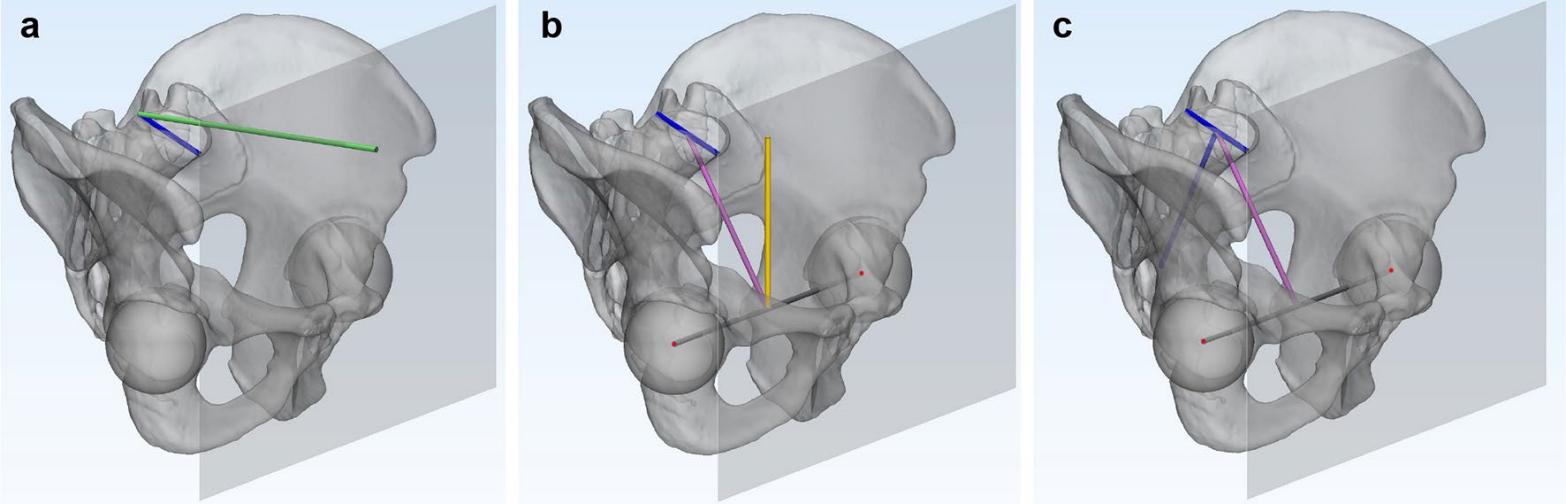
Representation of 3D pelvic parameters. The transparent grey pelvis indicates a pelvis in a random position. The transparent grey plane indicates a vertical plane parallel to the bicoxofemoral axis. The red dots indicate the centers of both femoral heads. The green bar indicates the normal axis of the vertical plane. The yellow bar indicates the vertical axis. The short blue bar indicates the line connecting the most anterior and posterior points of the sacral upper endplate. The long blue bar is a line perpendicular to the short blue bar. The pink bar connects the midpoint of the blue bar and the midpoint of the bicoxofemoral axis. MIMICS 20.0 (Materialise, Leuven, Belgium) and 3-matic 12 (Materialise) were used to create the image. (**a**) The sacral slope is the angle between the green and blue bars. (**b**) The spinopelvic tilt is the angle between the pink and yellow bars. (**c**) The pelvic incidence is the angle between the long blue and pink bars.

The three pelvic parameters generally investigated include sacral slope (SS), spinopelvic tilt (sPT), and pelvic incidence (PI)^3,11,17^. The SS was defined as the angle in the hip sagittal plane between the sacral upper endplate and the hip axial axis. The sPT was defined as the angle in the hip sagittal plane between the hip frontal plane and the line connecting the midpoint of the sacral plate and the bicoxofemoral axis. The sPT was considered positive when the sacral endplate moved forward. The PI was defined as the angle in the hip sagittal plane between the line perpendicular to the sacral plate, at its midpoint, and the line connecting the midpoint of the sacral plate and the bicoxofemoral axis (Fig. 5).

### Pelvic phantom

According to the description of pelvic orientation, an independent researcher (JTK), who was not one of the observers, devised a positional device that visualized the position of a symmetric pelvic model.

#### Symmetric pelvis model

A set of CT images of the hip of a 22-year-old man with suspected osteoid osteoma on the left femur neck was used to design a completely symmetric hip model containing the pelvis and proximal femur.

The CT scan of the pelvis and proximal femur was reconstructed into a 3D model using MIMICS 20.0 (Materialise, Leuven, Belgium). The right hemipelvis, containing the proximal femur, was mirrored to the pelvic sagittal plane and fused to the right hemipelvis itself to form a symmetric hip model^45^. The distance from the center of the femoral head to the sagittal plane was measured using 3-matic modeling software (Materialise, Leuven, Belgium) at 80.23 mm.

The 3D image was 3D printed (Projet360, 3D Systems Inc., Rock Hill, CA, USA) using plaster material that absorbs radiation (VisiJet PXL, 3D Systems Inc.). The accuracy of the output was within 100 μm, according to the information provided by the manufacturer.

#### Positioning device

A positioning device (Yes-protec, Dong-Tan, Korea) was designed to control the orientation of the symmetric pelvis model according to the defined sequence of rotation, according to the globographic coordinate system. It was made from radiolucent material to not interrupt the projection of the pelvic model.

The margins of error for positioning the device were 0.138° for flexion, 0.225° for obliquity, and 0.191° for rotation, as the thickness of marking was 0.6 mm, 1 mm, and 0.5 mm, and the radius of the positioning device was 250 mm, 255 mm, and 150 mm, respectively.

### Imaging and measurements

Biplanar radiographic acquisitions were performed using the EOS (EOS Imaging, Paris, France), which was equipped with aluminum and copper beam filters. The pedestal of the EOS was confirmed to be flat using a levelling machine.

#### Acquisitions

The image acquisition protocol of ‘pelvis morphotype 1’ adopts an aluminum spectral filter for both frontal and lateral tubes to reproduce the real imaging context of the pelvis^44^.

The center of rotation was placed at the midpoint of the bicoxofemoral axis for the center of the detectors to be in perfect alignment with the center of rotation.

**Figure 6:**
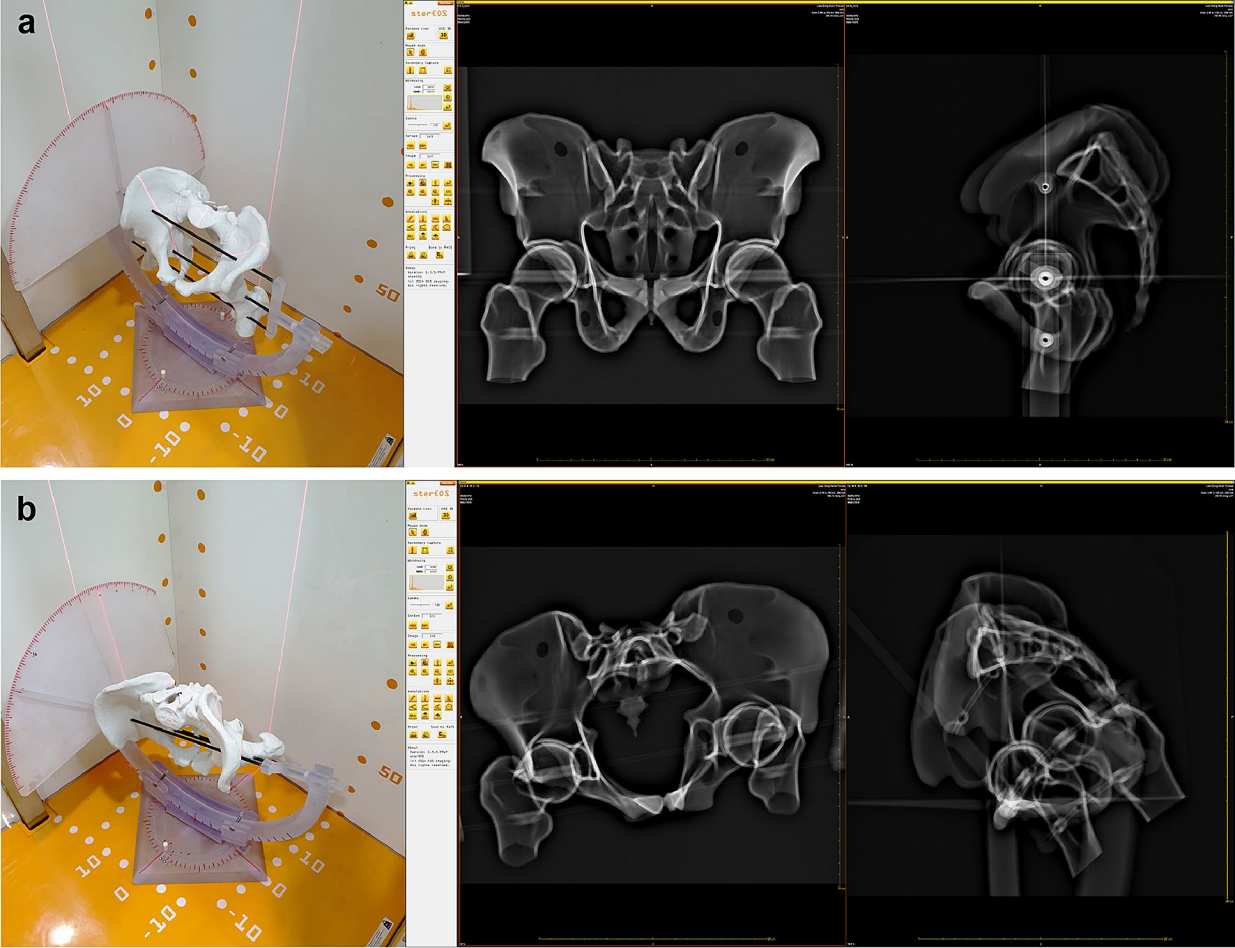
Image acquisition of the pelvic phantom performed for 105 position variations. SterEOS software (version 1.5.3.7947, EOS Imaging, Paris, France) was used to create the image. (**a**) The biplanar images depict the neutrally positioned phantom in the EOS system. (**b**) The biplanar images reveal the phantom with + 10° rotation and − 10° obliquity, and 45° flexion.

Variation in orientation consisted of 3 variations in obliquity, 7 variations in flexion, and 5 variations in rotation, resulting in 105 pairs of anteroposterior and lateral scanograms. The range and interval were from − 10° to 10° of obliquity, with increments of 10°; from − 45° to 45° of flexion, with increments of 15°; and from − 20° to 20° of rotation, with increments of 10° (Fig. 6).

#### Measurements

With the use of SterEOS software (version 1.5.3.7947, EOS Imaging), the manual registration of anatomical landmarks, such as both femoral heads, upper endplate of the sacrum, both sacroiliac joints, both anterior superior iliac spines (ASISs), and the midpoint of both pubic tubercles on the biplanar radiographic images enabled the semiautomated measurement of pelvic parameters and the orientation of the pelvis. Three different observers, including an orthopedic surgeon, with 7 years of experience, and two musculoskeletal radiologists, with a minimum of 7 years of experience, underwent a 1-day training session with 20 samples provided by the study designer. In the training session, the methods of measurements were standardized as below. The midpoint of both pubic tubercles was used to represent both these structures; the points on both ASISs, which met a line tangential to both these structures and the midpoint of both pubic tubercles, were used to represent both ASISs.

No numerical feedback was provided during manual registration, as inherent to the sterEOS system design. Thus, the raters could only adjust the registration before acquisition of numerical results. Once the result was obtained at the final step of each measurement, no adjustment of registration or remeasuring was allowed. The measurements of the 105 pairs of images were made by the three raters, separately.

### Statistical analysis

Trueness, which is defined as the closeness of agreement between the average of repeated measurements and the reference value, refers to a systematic error. It is quantified using the measurement bias (δ)^46^.

$$\updelta = reference\;value - measurements $$

However, as negative measurement errors cannot offset positive measurement errors, the arithmetic mean partially reflects the trueness of measurements. Thus, the standard deviations of bias were used as the representative values of trueness.

The rates of measurement errors outside of the positioning error margin (RMEPEM) of the pelvic position were evaluated based on the error margins of the positioning device.

$$ {\text{RMEPEM}} = \frac{Number\;of\;measurements\;out\;of\;positioning\;error\;margin}{{Number\;of\;all \;the\;measurements}} $$

Precision, which is defined as the closeness of the results obtained by replicate measurements made by multiple operators, refers to a random error^47^. It is often assessed by calculating the intraclass correlation coefficient (ICC). Thus, the ICC (3, k) model for consistency was calculated for each parameter, at each position, for the whole data set.

However, the ICC is highly dependent on the distribution of subjects, and it does not provide results directly related to the quantified uncertainty of measurement^48,49^. A single pelvic phantom, which restricts morphometric parameters such as PI to single values, was used in the present study; thus, the reliability of PI could not be measured using ICC values.

Therefore, precision was also assessed according to the guidelines of the ISO 5725-2 standard, using the root-mean square average of the standard deviations of each case (RMS_SD_)^47,50^.

$$ {\text{RMS}}_{{{\text{SD}}}} :{ }\sqrt {\sum \left( {\frac{SD\;case}{{Number\;of\;cases}}} \right)} = \sqrt {\sum \left( {\frac{{\sum \frac{{\left( {Reference\;value - measurements} \right)^{2} }}{Repeated\;measurements - 1}}}{Number\;of\;cases}} \right)} $$

This approach allows estimation of a 95% confidence interval for the position precision provided by ± 2 RMS_SD_.

The global uncertainty value (± ε) includes both trueness and precision. It was calculated as the sum of the standard deviation of bias (${\text{SD}}_{\updelta }$) and 95% confidence interval (2 RMS_SD_).

$$ {\text{Global uncertainty}}\,(\upvarepsilon ) = {\text{SD}}_{\updelta } + 2RMS_{SD} $$

The relationship between pelvic orientation variation, which was represented as the sum of flexion, obliquity, and rotation of the pelvis, and the sum of the differences between the reference value and measured values was analyzed using non-parametric locally estimated regression (local polynomial regression [LOESS]) analysis^51^.

As these data contain 4-dimensional information, the pattern of measurement errors was depicted on multiple heat maps.

The effect model of pelvic orientation on the measurement error was established based on the heat maps; the model was analyzed using Spearman’s correlation.

All statistical analyses were performed with R software (www.r-project.org), version 3.6.1. and Microsoft Excel (Microsoft Corp., Redmond, WA, USA)^52^. *p* < 0.05 was considered to indicate statistical significance.
